# Children and young people with persistent post-COVID-19 condition over 24 months: a mixed-methods study

**DOI:** 10.1136/bmjpo-2025-003634

**Published:** 2025-10-21

**Authors:** Esther Ortega-Martin, Alvin Richards-Belle, Fiona Newlands, Roz Shafran, Terence Stephenson, Natalia Rojas, Neha Batura, Marta Buszewicz, Emma Dalrymple, Isobel Heyman, CLoCk Consortium, Anthony Harnden, Snehal M Pinto Pereira

**Affiliations:** 1 Department of General Economy (Health Sociology area), University of Cadiz, Cádiz, Spain; 2 Division of Surgery & Interventional Science, University College London, London, UK; 3 Division of Psychiatry, University College London, London, UK; 4 UCL Great Ormond Street Institute of Child Health, University College London, London, UK; 5 Institute for Global Health, University College London, London, UK

**Keywords:** COVID-19, Adolescent Health, Health services research, Qualitative research, Child Health

## Abstract

**Purpose:**

While most children and young people (CYP) recover from COVID-19, some develop ‘post-COVID-19 condition’ (PCC), affecting their health and well-being. We explored (1) whether distinct persistent PCC symptom subgroups exist in CYP and whether these subgroups remain stable up to 24 months postinfection; (2) whether impairments differ across subgroups and (3) how CYP with persistent PCC describe the evolving impact of the pandemic/lockdowns on their health and experiences up to 24 months postinfection.

**Methods:**

A cohort of CYP across England was recruited in 2020–2021 (the children and young people with Long COVID study). A subsample of 68 CYP meeting the PCC Delphi research definition at 3, 6, 12 and 24 months post-PCR-confirmed infection was analysed. Latent class analysis identified symptom subgroups (objective 1); associations with impairments (measured via EuroQol Five Dimensions Youth) were examined (objective 2). Free-text responses from six CYP at all four follow-up points (n=24) were thematically analysed to capture evolving experiences (objective 3).

**Results:**

Included CYP were older (72.1% were 15–17 years), female (82.4%) and white (80.9%). Two symptom groups emerged: a frequent symptom subgroup (median: 6.5–9 symptoms over time, mainly shortness of breath and tiredness); and a less frequent symptom subgroup (median: 4–5 symptoms, mostly tiredness). Generally, no association was found between symptom subgroups and impairments. Qualitative analysis indicated feelings of anxiety, respiratory problems and concerns around relaxation of lockdown restrictions persisted over follow-up. School-related worries were transient.

**Discussion:**

Even CYP with persistent PCC characterised by fewer symptoms experience long-term anxiety and impact, emphasising even few symptoms can be debilitating and underscoring the need for personalised PCC management for CYP.

**WHAT IS ALREADY KNOWN ON THIS TOPIC**

- Some children and young people experience persistent post-COVID-19 condition, but long-term symptom profiles and impact are unclear.
- Most existing studies are short-term and lack integration of children and young people’s own narratives over time.

**WHAT THIS STUDY ADDS**

- Over time, two distinct symptom subgroups were identified among children and young people with persistent post-COVID-19 condition.
- Even children and young people with fewer symptoms report long-lasting anxiety and emotional distress.
- Feelings of anxiety, respiratory problems and concerns around relaxation of lockdown restrictions persisted over follow-up; school-related worries were transient.

**HOW THIS STUDY MIGHT AFFECT RESEARCH, PRACTICE OR POLICY**

- Findings emphasise that, regardless of number of symptoms experienced, personalised care pathways for children and young people with persistent post-COVID-19 condition are needed.
- We illustrate the importance of taking a longitudinal mixed-methods approach to understand chronic conditions in children and young people.
- Findings could inform service planning by underscoring the long-term psychological impact of persistent post-COVID-19 condition and pandemic-related disruptions.

## Introduction

 By March 2023, 676 million people worldwide had contracted SARS-CoV-2.^1^ While most recover, some continue to experience persistent symptoms for months after acute infection^2^—a phenomenon termed ‘Long COVID’ or ‘post-COVID-19 condition’ (PCC). Estimates of PCC prevalence vary widely, ranging from 6% to 30% in adults,^3 4^ and 4% to 66% in children and young people (CYP).^5 6^ Understanding PCC in CYP is particularly important,^6^,^8^ as childhood and adolescence are critical developmental periods when socialisation and friendships play a key role in mental health, brain development and self-conceptualisation.^9^ PCC symptomology often includes shortness of breath (SOB), fatigue, problems with sleep, affective symptoms and headache.^10^ These symptoms can affect young people’s ability to carry out daily activities, socialise with friends/family and attend school. Thus, PCC can impact CYPs’ immediate and longer-term health, well-being and quality of life.^10^

Studying PCC in CYP presents at least four notable challenges. First, PCC definitions are inconsistently used in the literature,^7 11^ making cross-study comparisons difficult. For example, discrepancies in study designs and conceptual frameworks may have led to inconsistent findings on quality of life of CYP with PCC.^12^ Second, with few exceptions,^13^ most studies are limited by short follow-up periods, hindering a comprehensive understanding of long-term impacts on health, well-being and quality of life.^14^ Third, while definitions of PCC in CYP acknowledge that symptoms may fluctuate,^15 16^ the definitions lack specificity regarding types of symptoms experienced. This leads to unanswered questions such as: Do distinct PCC symptom clusters exist among CYP? And do these clusters remain stable over time? Finally, more quantitative than qualitative evidence has been collected on PCC.^17^,^19^ In particular, CYP’s descriptions of their health and experiences during the pandemic have been little studied,^20^ despite the importance of documenting/integrating their experiences into research findings.^21^

To address these challenges, we aimed to improve understanding of symptom profiles of CYP with persistent PCC and examine how PCC impacts their lives. Using a mixed-methods approach, we set out to answer the following three questions: (1) Do distinct symptom subgroups exist among CYP with persistent PCC, and do these subgroups remain stable at 3, 6, 12 and 24 months post-SARS-CoV-2 infection? (2) Do reported impairments differ between symptom subgroups? and (3) How do CYP with persistent PCC describe the evolving impact of the pandemic and lockdowns on their health and experiences over a 24-month period?

This study was explicitly designed as an exploratory investigation to provide in-depth understanding of symptom subgroups, impairments and lived experiences of CYP who persistently meet the research definition of PCC over a 24-month period. Importantly, a comparator group of CYP who did not persistently meet the PCC definition was not included, as the focus was on capturing and understanding experiences of those persistently affected.

## Methods

We used a subsample of data from the Children and young people with Long COVID (CLoCk) study^22^ (which is now publicly available^23^). This is a cohort of CYP living in England aged 11–17 years when they PCR-tested for SARS-CoV-2 between September 2020 and March 2021. The number of data collection sweeps varied depending on the CYP’s month of PCR testing. This study focused on CYP who PCR-tested positive for SARS-CoV-2 in January, February or March 2021; 23 048 test-positive CYP were invited to participate and 3321 enrolled. Of these, 943 CYP provided data at 3, 6, 12 and 24 months post-testing, with 68 persistently meeting the PCC definition (see below) at all four time points.^13^

###  Research team and guidelines

The research team included clinicians, psychologists, mixed methods and quantitative experts. Our work follows the Mixed Methods Reporting in Rehabilitation & Health Sciences guidelines.^24^

### Measures

The UK Health Security Agency provided data on sex at birth, age at PCR-testing, region of residence and the 2019 English Index of Multiple Deprivation (IMD; used as a proxy for socioeconomic status^25^). CYP reported their ethnicity at study enrolment (3 months post-testing in the examined subsample). At all data collection sweeps, symptoms currently being experienced were self-reported. The symptom list was the same at all time points, with the addition of ‘problems sleeping’ at the 12 and 24 months data collections.^22^ The symptom list broadly aligned with the International Severe Acute Respiratory and Emerging Infection Consortium Paediatric COVID-19 questionnaire.^26^ Validated scales, including the EuroQol Five Dimensions Youth (EQ-5D-Y) questionnaire^27^ (a measure of health-related quality of life) and the Strengths and Difficulties Questionnaire^28^ (SDQ; used to measure emotional and behavioural difficulties), were also collected. There was always a final optional question that prompted CYP to tell us about their health or how the pandemic/lockdown affected them; responses were truncated at 621 characters.

### Defining persistent PCC

At all data collection sweeps post-positive SARS-CoV-2 PCR test, the published PCC Delphi research definition^15^ was operationalised as explained elsewhere.^13^ Briefly, PCC was defined as (1) experiencing ≥1 symptom from the prespecified symptom list (described above) and (2) some or a lot of problems with mobility, self-care, doing usual activities, having pain/discomfort or feeling very worried/sad/unhappy (measured via EQ-5D-Y^27^). CYP were considered to have persistent PCC if they PCR-tested positive for SARS-CoV-2 between January and March 2021 and met the PCC Delphi research definition at every data collection point (ie, at 3, 6, 12 and 24 months post-infection).

### Quantitative analysis

To determine the representativeness of our analytical sample, we examined whether CYP (following a positive PCR test) who persistently met the PCC definition over 24 months differed from those who never met the PCC definition over the same timeframe. We used χ^2^, Fisher’s exact or Kruskal-Wallis rank tests as appropriate.

We used a latent class approach^29^ to determine whether there were distinct subgroups of CYP with persistent PCC reporting different clusters of symptoms (question 1). At each time point, we fitted latent class models with a 2, 3 and 4-class solution. We selected the best class solution by choosing the model with the lowest Akaike information criterion (AIC). When the difference between two AIC values was <4, it was deemed negligible, and the model with fewer classes was chosen. Predicted class membership was estimated^29^ and used to assign CYP to their most likely class. We describe characteristics of CYP in each latent class in terms of symptoms experienced.

We examined impairments reported by CYP with persistent PCC by identified latent classes (question 2). Specifically, we examined the proportion of CYP reporting impairments from the EQ-5D-Y scale at each time point. As several cell frequencies were low (<5), we used Fisher’s exact test to determine if there was an association between impairments and latent class membership. Data management and analyses were carried out in R (V.4.4.1),^30^ using the poLCA (V.1.6.0.1) package^31^ for the latent class analysis.

#### Qualitative analysis

Aiming to explore evolving perceptions and experiences of CYP with persistent PCC up to 24 months post-SARS-CoV-2 infection (question 3), we conducted a reflexive thematic analysis^32^ on the subset of CYP who provided free-text responses at all four follow-up time points. Data coding involved an iterative review in which EO applied descriptive codes to each meaningful unit of text. Codes were grouped into broader categories and refined into related themes. The initial thematic scheme was revised following discussions with coauthors (AR-B, FN, RS, TS, NR and SMPP), ensuring a comprehensive representation of participants’ experiences. Qualitative analyses were carried out in ATLAS.ti (V.24.1.1).^33^

### Integration of quantitative and qualitative analyses

**Figure 1:**
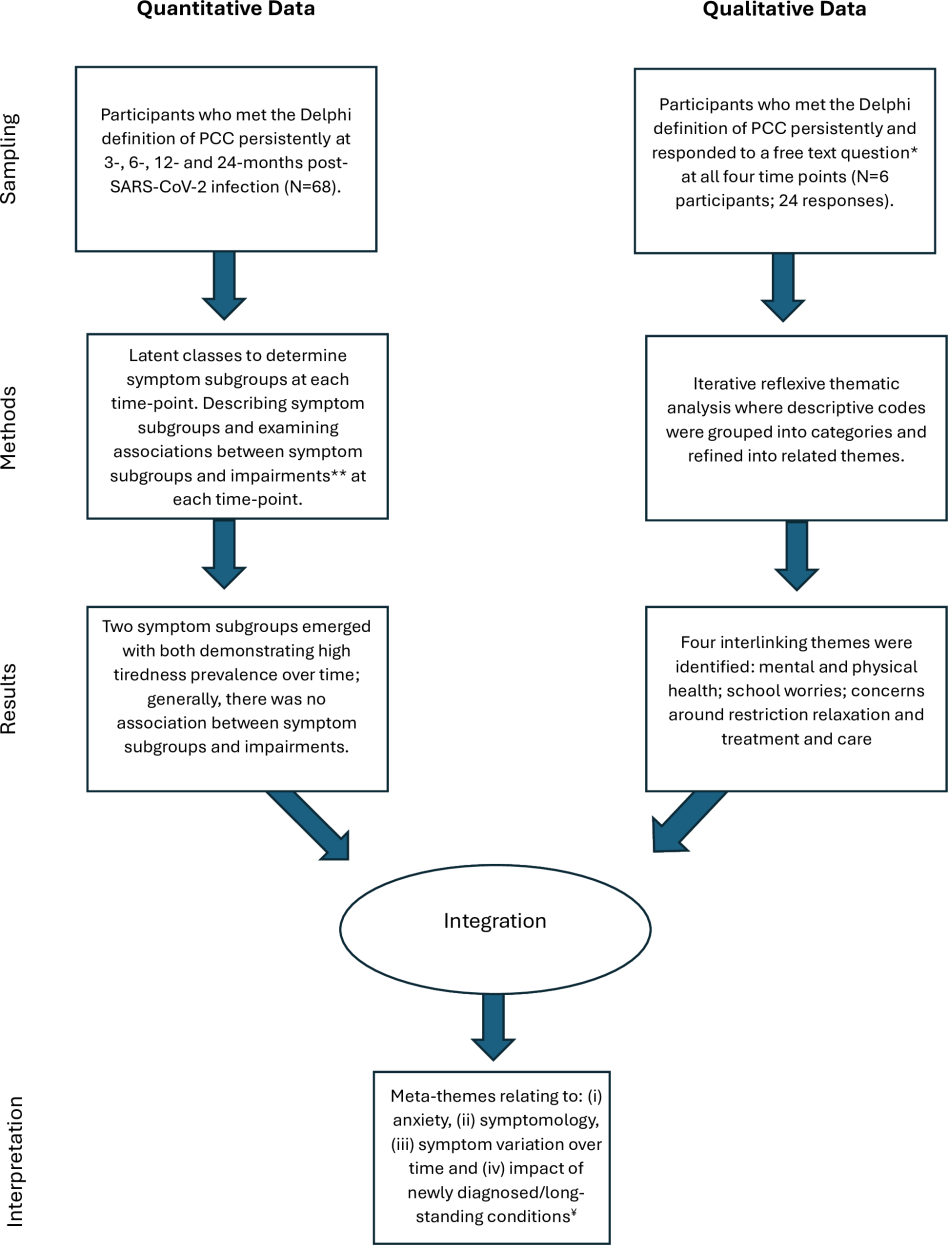
Application of the parallel mixed-methods design. *The question stated: “…use this space if there is anything else you would like to tell us about your health or how the pandemic or lockdown have affected you”; **As assessed on the EQ-5D-Y scale; ¥see results and table 3 for details of each meta-theme. EQ-5D-Y, EuroQol 5 Dimensions Youth; PCC, post-COVID-19 condition.

Our quantitative and qualitative data were collected and examined at the same time. Thus, to integrate our quantitative and qualitative findings, we applied a convergent parallel mixed methods design^34^ as illustrated in figure 1. This data integration approach allows us to identify meta-themes by determining potential areas of similarities and differences in CYP’s experiences of persistent PCC across the different data sources.

### Patient and public involvement

This study is based on data collected as part of the CLoCk study, which actively integrated the participation of CYP in all phases of the research process. For example, CYP were involved in developing the Delphi research definition of PCC and co-developing communication and dissemination strategies. Therefore, although patients and the public were not directly involved in the specific design or conduct of analysis undertaken in this manuscript, their prior contributions were essential in influencing the methodological and conceptual underpinnings of our work.

## Results

68 CYP from the CLoCk study PCR-testing positive between January and March 2021 met the PCC Delphi research definition persistently at 3, 6, 12 and 24 months and were included in the quantitative analysis (table 1). Most of these CYP were older at PCR testing (72.1% were 15–17 years), female (82.4%) and of white ethnicity (80.9%). Compared with CYP who provided data at all four time points but never met the PCC definition, the analytical sample was older and more likely to be female (online supplemental table 1). The two groups were otherwise similar in terms of deprivation (IMD quintile), region of residence and ethnicity. As expected, the two groups differed in the number of symptoms reported from 3 to 24 months, as well as in the prevalence of SDQ ‘caseness’; CYP who persistently met the PCC definition were more likely to report a higher symptom burden and to exhibit a more adverse mental health profile (online supplemental table 1).

**Table 1:** Characteristics (N (%)) of PCR-test positive CYP persistently meeting the PCC research definition at 3, 6, 12 and 24 months post-testing included in the quantitative analysis (N=68)

Age at PCR testing (years)	
11–14	19 (27.9)
15–17	49 (72.1)
Sex at birth	
Female	56 (82.4)
Male	12 (17.6)
IMD quintile	
1 (Most deprived)	10 (14.7)
2	16 (23.5)
3	18 (26.5)
4	12 (17.6)
5 (least deprived)	12 (17.6)
Region of residence	
Southeast	13 (19.1)
London	12 (17.6)
Southwest	12 (17.6)
East of England	10 (14.7)
West Midlands	8 (11.8)
Northwest	6 (8.8)
Northeast	1 (1.5)
East Midlands	4 (5.9)
Yorkshire and The Humber	2 (2.9)
Ethnicity	
White	55 (80.9)
Asian/Asian British	7 (10.3)
Mixed/other	5 (7.4)
Black/African/Caribbean	1 (1.5)

CYP, children and young people; IMD, Index of Multiple Deprivation; PCC, post-COVID-19 condition.

### Symptom profiles at 3, 6, 12 and 24 months post-SARS-CoV-2 infection

**Figure 2:**
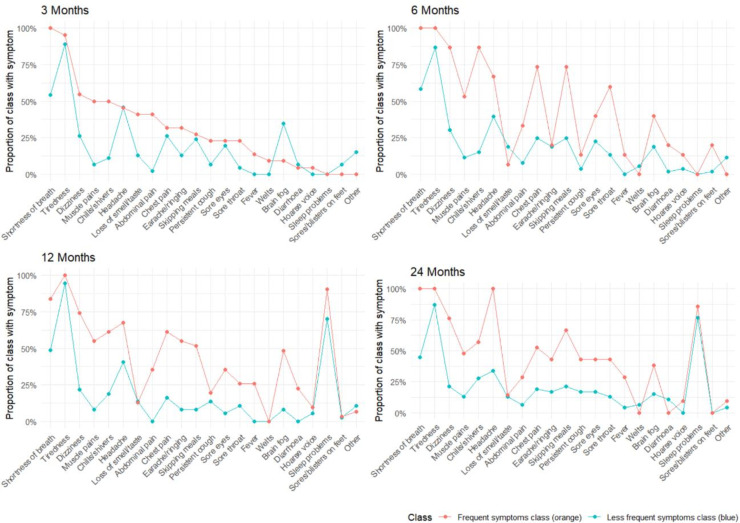
Proportion of each latent class reporting specific symptoms at each follow-up time point.* *N (%) of CYP with persistent PCC in the frequent symptom class: 22 (32.4%) at 3 months, 15 (22.1%) at 6 months, 31 (45.6%) at 12 months and 21 (30.9%) at 24 months; N (%) of CYP with persistent PCC in the less frequent symptom class: 46 (67.7%) at 3 months, 53 (77.9%) at 6 months, 37 (54.4%) at 12 months and 47 (69.1%) at 24 months. NB ‘sleep problems’ only asked about at 12 and 24-months. CYC, children and young people; PCC, post-COVID-19 condition.

At all four time points, there were two distinct symptom profile groups: a group with frequent symptoms and a group with less frequent symptoms (figure 2). The group with frequent symptoms reported a median (IQR) of 6.5 (5, 7.75) symptoms at 3 months; 9 (8, 10) symptoms at 6 months; 9 (8, 11) symptoms at 12 months; and 9 (9, 12) symptoms at 24 months post-infection. The median (IQR) number of symptoms in the less frequent symptom group was: 4 (3, 5) at 3, 6 and 12 months, and 5 (3.5, 6) at 24 months post-infection. At all time points, there were fewer CYP in the group with frequent symptoms versus the group with less frequent symptoms. CYP in the frequent symptoms group ranged from 15 (22%) at 6 months to 31 (46%) at 12 months, while CYP in the less frequent symptoms group ranged from 37 (54%) at 12 months to 53 (78%) at 6 months.

The frequent symptom group was dominated by ‘unusual fatigue/tiredness’ and ‘unusual SOB’ (referred to here as tiredness and SOB, respectively; figure 2). The prevalence of tiredness in the frequent symptom group varied between 95% (3 months) to 100% (6, 12 and 24 months), and SOB prevalences varied between 84% (12 months) to 100% (3, 6 and 24 months). Symptoms like chills and headache featured at some, but not all, time points: chills varied between 50% (3 months) to 87% (6 months); headache from 45% (3 months) to 100% (24 months).

At all four time points, CYP in the less frequent symptom group also experienced tiredness but fewer CYP experienced SOB. In this group, the prevalence of tiredness ranged from 87% (6 and 24 months) to 95% (12 months) and SOB ranged from 45% (24 months) to 59% (6 months).

Although only asked at 12 and 24 months, problems sleeping were notable in both subgroups at 12 months and 24 months, affecting 90% of the frequent symptom group and 70% of the less frequent symptom group at 12 months and 86% and 77%, respectively, at 24 months.

24 (35.3%) CYP were always in the less frequent symptom subgroup and 3 (4.4%) were always in the frequent symptom subgroup; the remaining 41 (60.3%) CYP moved between the two subgroups over time.

### Reported impairments by symptoms subgroup

In general, there was no association between identified symptom subgroups and problems with mobility, doing usual activities and self-care (as measured via the EQ-5D-Y questionnaire) at all examined time points (table 2). However, at almost all time points, most CYP belonging to the frequent symptom subgroup were in some or lots of pain, with statistically significant differences from the less frequent symptom subgroup at 6 and 12 months. Similarly, at almost all time points, most CYP belonging to the frequent symptom subgroup were a bit or very worried/sad/unhappy (with statistically significant differences from the less frequent symptom subgroup at 12 months).

**Table 2:** Distribution of individual EQ-5D-Y domain items at 3, 6, 12 and 24 months post-SARS-CoV-2 infection, by symptom subgroups*, in CYP who persistently meet the PCC research definition (N (%))

Symptom group	3 months		6 months		12 months		24 months	
	Less frequent (N=46)	Frequent (N=22)	Less frequent (N=53)	Frequent (N=15)	Less frequent (N=37)	Frequent (N=31)	Less frequent (N=47)	Frequent (N=21)
Problems with:								
Mobility (walking)								
None	27 (58.7)	17 (77.3)	37 (69.8)	8 (53.3)	21 (56.8)	20 (64.5)	30 (63.8)	11 (52.4)
Some	19 (41.3)	5 (22.7)	15 (28.3)	7 (46.7)	14 (37.8)	11 (35.5)	14 (29.8)	10 (47.6)
Lots	0 (0)	0 (0)	1 (1.9)	0 (0)	2 (5.4)	0 (0)	3 (6.4)	0 (0.0)
Association between symptom group and impairment: p value†	0.18		0.39		0.59		0.24	
Look after self (wash/dress)								
None	38 (82.6)	19 (86.4)	46 (86.8)	11 (73.3)	32 (86.5)	25 (80.7)	33 (70.2)	18 (85.7)
Some	8 (17.4)	3 (13.6)	7 (13.2)	4 (26.7)	4 (10.8)	5 (16.1)	13 (27.7)	1 (4.8)
Lots	0 (0)	0 (0)	0 (0)	0 (0)	1 (2.7)	1 (3.2)	1 (2.1)	2 (9.5)
Association between symptom group and impairment: p value†	1.00		0.24		0.86		0.04	
Doing usual activities								
None	16 (34.8)	5 (22.7)	21 (39.6)	3 (20.0)	11 (29.7)	11 (35.5)	13 (27.7)	4 (19.1)
Some	25 (54.4)	13 (59.1)	29 (54.7)	11 (73.3)	23 (62.2)	15 (48.4)	32 (68.1)	16 (76.2)
Lots	5 (10.9)	4 (18.2)	3 (5.7)	1 (6.7)	3 (8.1)	5 (16.1)	2 (4.3)	1 (4.8)
Association between symptom group and impairment: p value†	0.54		0.34		0.45		0.80	
Pain/discomfort								
None	15 (32.6)	5 (22.7)	16 (30.2)	0 (0)	17 (46.0)	2 (6.5)	10 (21.3)	1 (4.8)
Some	27 (58.7)	12 (54.6)	35 (66.0)	12 (80.0)	18 (48.7)	24 (77.4)	33 (70.2)	15 (71.4)
Lots	4 (8.7)	5 (22.7)	2 (3.8)	3 (20.0)	2 (5.4)	5 (16.1)	4 (8.5)	5 (23.8)
Association between symptom group and impairment: p value†	0.25		<0.01		<0.01		0.10	
Feeling worried/ sad/unhappy								
No	9 (19.6)	2 (9.1)	7 (13.2)	1 (6.7)	10 (27.0)	0 (0)	4 (8.5)	0 (0)
A bit	22 (47.8)	9 (40.9)	26 (49.1)	7 (46.7)	16 (43.2)	12 (38.7)	24 (51.1)	12 (57.1)
Very	15 (32.6)	11 (50.0)	20 (37.7)	7 (46.7)	11 (29.7)	19 (61.3)	19 (40.4)	9 (42.9)
Association between symptom group and impairment: p value†	0.35		0.78		<0.01		0.54	

* Symptom subgroups identified by latent class analysis at each time point (see methods).

† From Fisher’s exact test.

CYP, children and young people; EQ-5D-Y, EuroQol 5 Dimensions Youth; PCC, post-COVID-19 condition.

### Free-text responses from CYP with persistent PCC from 3 to 24 months postinfection

Six CYP provided free-text responses at all time-points (n=24 responses). Most of these CYP were older at PCR testing (15–17 years n=5 vs 11–14 years n=1), all were female, and all but one were of White ethnicity. Of these six CYP, two were always in the less frequent symptom subgroup, while one was always in the frequent symptom subgroup. We indicate symptom subgroup membership by using ‘H’ and ‘L’ with formatting to indicate the timing of the quote. For example, ‘HLH**H**’ refers to the participant who was in the frequent symptom subgroup at all but the 6-month time point and refers to a quote made 24 months postinfection.

Four interlinking themes were identified and illustrated below.

#### Mental and physical health

All CYP thought the pandemic and lockdowns affected their mental and/or physical health. Their experiences highlighted an increase in mental health problems, especially anxiety, with some discussing a depression diagnosis, while others described feelings of depression:

It’s definitely made me more anxious than I used to be as I have been alone for a long period of time (**H**LLL)

For this young person, levels of anxiety did not improve over time:

[…] Higher social anxiety levels. Been feeling a lot lower mentally (H**L**LL)

Feelings of anxiety that persisted over follow-up were not limited to those in the frequent symptom class. One person who was always in the less frequent symptom class reported

Since … the start of the pandemic my anxiety levels have increased greatly. (**L**LLL).

Similar comments were made throughout follow-up; for example, at 6 months, the same person commented on the impact anxiety had on their life:

I still struggle with anxiety around covid … it is affecting my family and other relationships. (L**L**LL).

And at 12 months:

I have been experiencing a lot of anxiety … it has stopped me from going out and doing things even when restrictions are eased (LL**L**L).

Over time, the root cause of experienced anxiety was queried. For example, 3 months post-testing, one person said:

it worsened my mental health by quite a lot. (**H**LHH).

By 12 months, they queried whether their ongoing anxiety was due to a positive COVID test or the pandemic more broadly:

My mental health has gotten considerably worse… I’m not sure if it has anything to do with a positive covid diagnosis but the covid pandemic hasn’t helped. (HL**H**H).

Respiratory problems were mentioned often by CYP in both symptom groups:

COVID and the pandemic has definitely affected my memory, my breathing and my mental health in negative ways (LLL**L**)

They were also mentioned repeatedly over time; for example, one person said at 3 months:

After having COVID … my chest was constantly tight and I struggled breathing just walking up the stairs. (**L**HHH)

Making similar comments throughout the follow-up:

Since I got covid, my breathing has been absolutely awful (L**H**HH).

Although not explicitly stated, only this person intimated symptoms waxed and waned over time:

Since having covid my health has been an up and down thing (LH**H**H)

CYP also mentioned a range of (unrelated) life events/diagnoses that occurred or were exacerbated during the pandemic that contributed to their symptoms:

I have a tumour … and I'm on medication which is gonna make me feel more drowsy and fatigued. (HL**L**L)

Since covid, my asthma has gotten worse. (HH**H**H).

Tiredness and headache were not mentioned in the free-text responses we reviewed.

#### School worries

CYP mentioned academic pressure caused by disruption to their routines (with subsequent impact on their mental health):

It’s impacted my grades as learning in lockdown, took it out of me … (**H**HHH)

The pressure from schoolwork … has definitely not helped (**L**LLL).

These worries were reported almost exclusively 3 months post-infection with no mention of school worries 12 and 24 months post-infection.

#### Concerns around restriction relaxation

CYP were concerned about lifting of restrictions and this view did not alter over time (at least until 12 months post-infection). For example, 6 months post-infection one person said:

I find it difficult going into shops where there are lots of people. This has been made harder to cope with because masks are not mandatory now. (H**H**HH)

And 12 months post-infection they said:

I would feel safer if masks were still mandatory in shops and school/college corridors. (HH**H**H)

Children in the less frequent symptom class were also concerned about restriction relaxation:

I have been experiencing a lot of anxiety since the start of the pandemic and it has stopped me from going out and doing things even when restrictions are eased. (LL**L**L).

#### Treatment and care

Treatments and clinical pathways of care for ongoing symptoms were mentioned, almost exclusively, 24 months post-infection:

Since having covid for the third time …, I have been referred to a post Covid clinic and [I am] now under the care of a paediatrics post Covid team (LHH**H**).

Some CYP discussed referrals to multiple specialists and difficulty with diagnosis:

I’ve had to do & continuing a lot of tests by my doctor to figure out why I’m physically feeling the way I’ve felt. I’ve been with the long Covid team, with the rheumatology team & waiting for the respiratory team. (HHH**H**).

### Identification of meta-themes by data integration

Table 3 and figure 1 summarise four meta-themes identified by the quantitative and qualitative findings and highlight areas of convergence and divergence. Table 3 also summarises key considerations when interpreting these results.

**Table 3:** Meta-themes identified from the quantitative and qualitative results

Meta-theme	Quantitative (N=68)	Qualitative* (N=24 responses from 6 CYP)	Quantitative and qualitative findings converge or diverge?	Considerations
Long-lasting anxiety experienced by CYP with persistent PCC	Many CYP (range: 38% at 3 months to 44% at 12 months) reported they were very worried/ sad/unhappy throughout the 24-month period.	Almost all CYP reported anxiety which did not improve over time. "*I have been experiencing a lot of anxiety … it has stopped me from going out and doing things even when restrictions are eased*” (LL**L**L).	Convergence	Qualitative findings provided context for experienced anxiety; anxiety might be due, at least in part initially, to the negative impacts of the COVID-19 pandemic, infection and lockdowns on CYP’s education and, more long-term, concerns about restriction relaxation.
Core symptoms: tiredness and shortness of breath	Tiredness was reported often and with high prevalence. In the frequent symptom group, prevalence varied between 95% to 100%; in the less frequent symptom group, prevalence ranged from 87% to 95%. Unusual shortness of breath was reported often in the frequent symptom group (84% to 100%). In the less frequent symptom group, shortness of breath ranged from 45% to 59%.	Tiredness was not mentioned in the free-text responses reviewed. Respiratory problems were mentioned often (and repeatedly) by CYP. “*COVID and the pandemic has definitely affected my memory, my breathing and my mental health in negative ways*” (LLL**L**)	Divergence: regarding tiredness. Convergence: shortness of breath commonly reported.	CYP may have experienced tiredness but did not find it debilitating, or did not attribute it to COVID-19 or related restrictions. Tiredness may have been so common and persistent that CYP considered it a ‘normal’ and therefore did not mention it. Participants were not systematically asked about specific symptoms, so tiredness may not have been explicitly reported in qualitative data.
Symptom profiles vary over time.	In the frequent symptom group, symptoms like chills and headache featured at some, but not all, time points: chills varied between 50% and 87%; headache from 45% to 100%. In the less frequent symptom group, prevalence of shortness of breath ranged between 45% and 59%.	Variation over time was suggested by free-text responses: *“Since having covid my health has been an up and down thing”* (LH**H**H)	Convergence	
Impact of newly diagnosed or long-standing conditions	N/A	Medical events during the pandemic (e.g., tumour diagnosis) and/or previous conditions (e.g., asthma) might exacerbate reported symptoms. *“Since covid, my asthma has gotten worse.”* (HH**H**H). Burden in terms of treatment and clinical care pathways for ongoing symptoms (reported almost exclusively 24-months post-infection). "*Since having covid for the third time …, I have been referred to a post Covid clinic and [I am] now under the care of a paediatrics post Covid team*” (LHH**H**).	Neither: theme from qualitative findings that was not explored quantitively	

* We indicate symptom subgroup membership by using ‘H’ and ‘L’ with formatting to indicate the timing of the quote. For example, ‘HLH**H’** refers to a participant who was in the frequent symptom subgroup at all but the 6-month time point and refers to a quote made 24 months post-infection.

CYP, children and young people; N/A, not available.

First, both quantitative and qualitative findings converge on highlighting the long-lasting anxiety experienced by CYP with persistent PCC. While acknowledging the prevalence varied by symptom frequency subgroup, many CYP (ranging from 38% (n=26) at 3 months to 44% (n=30) at 12 months) reported they were very worried/sad/unhappy throughout the 24-month period. Qualitative findings corroborated quantitative observations with almost all CYP involved reporting anxiety which did not improve over time. The qualitative findings provided context around this anxiety, suggesting it might be due, at least in part initially, to the negative impacts of the COVID-19 pandemic, infection and lockdowns on CYP’s education and, more long-term, concerns about restriction relaxation.

Second, while quantitative and qualitative findings agreed on SOB being commonly reported, there was inconsistency regarding tiredness. Tiredness was reported often and with high prevalence quantitatively, but only mentioned once qualitatively (in relation to tumour medication side effects). Several factors may explain this discrepancy. It is possible that although CYP experienced tiredness, it was not perceived as debilitating or attributed to COVID infection/associated lockdowns. Alternatively, tiredness may have been so pervasive that it was normalised and thus not considered noteworthy. Finally, as participants were not systematically probed about specific symptoms, experiences of tiredness may not have been fully captured qualitatively.

Third, symptom profiles varied over time. For example, in the less frequent symptom subgroup, the prevalence of SOB ranged between 45% and 59%. In the frequent symptom subgroup, chills and dizziness were reported often at some, but not all, time points. Free text corroborated these observations.

Fourth, only the qualitative analysis highlighted that medical events during the pandemic (eg, tumour diagnosis) and/or previous conditions (eg, asthma) might exacerbate reported symptoms. Relatedly, the qualitative analysis indicated a burden in terms of treatment and clinical care pathways for ongoing symptoms, which were reported almost exclusively 24 months post-infection.

## Discussion

We found at multiple time points over a 2-year period, there were two distinct subgroups of CYP with persistent PCC: a frequent symptom and a less frequent symptom group. Although many CYP (n=24; 35.3%) were always in the less frequent symptom group, most (n=41; 60.3%) fluctuated between the two groups over time, with only 3 CYP always being in the high frequency symptom group. Importantly, CYP in both subgroups experienced multiple symptoms (median 6.5–9 symptoms in the frequent symptom subgroup; median 4–5 symptoms in the less frequent symptom subgroup). Regarding impairments, there was generally no association between symptom subgroups and problems with mobility, doing usual activities and self-care over the 2-year period. However, compared with the less frequent symptom subgroup, more CYP in the frequent symptom subgroup were in some/lots of pain and reported feeling a bit or very worried/sad/unhappy. The qualitative analysis demonstrated anxiety, respiratory problems and concerns around restriction relaxation persisted over follow-up, appearing independent of symptom frequency subgroup membership. School worries were transient and limited to the start of follow-up (ie, April–June 2021).

By adopting a mixed-methods approach, we provide a nuanced view of health and experiences of CYP with persistent PCC. Nonetheless, study limitations are acknowledged. Our sample size was relatively small (N=68 (quantitative); N=24 from 6 CYP (qualitative)), limiting our analysis power. While this may reflect the relative rarity of persistent PCC in CYP in the general population, generalisability of findings also warrants consideration. For example, our findings may not be applicable to other countries or other subgroups of CYP within England (eg, of Black/African/Caribbean ethnicity). Our qualitative analysis was limited to examining responses from CYP who filled in free text at all time points, and we acknowledge that this is a small, selective subgroup (all female, mostly older CYP and White ethnicity). Nonetheless, the sample does provide information on how the impact of the pandemic/lockdowns evolved over time. For example, from initial worries about schooling to concerns about treatment/care 24 months post-infection. A more detailed cross-sectional analysis of a larger number of free-text responses is available elsewhere,^20^ as is a richer qualitative analysis from direct interviews.^35^ However, the added value of this current manuscript is that we describe, in depth, symptom clusters, impairments, health and experiences of CYP persistently meeting the PCC definition over a 24-month period. Crucially, we also describe how these symptom clusters, impairments and experiences evolved over time. As the work presented here is exploratory, we did not compare our findings to a group of CYP who did not experience persistent PCC. Finally, while the online platform truncated free-text responses at 621 characters, this applied to only two (of the 24) responses examined.

Mixed-methods studies with repeat, long-term follow-up of persistent PCC in CYP are rare. However, our findings agree with and extend previous quantitative and qualitative work. Our quantitative finding that tiredness is a commonly reported persistent PCC symptom agrees with systematic reviews.^10 36^ Here we extend this observation to demonstrate that there are two different PCC symptom profile subgroups, both reporting high levels of tiredness. Tiredness was rarely mentioned in qualitative analysis, contrasting with previous studies.^35 37^ Discrepancies could be due to multiple reasons, including participants not being specifically probed about symptoms and thus descriptions of tiredness may not be fully captured qualitatively. Future studies should examine tiredness due to PCC in more detail, including its overlap with Chronic Fatigue Syndrome, and potentially assess postexertional malaise. Previous studies have identified PCC symptom clusters in adults^38 39^ but the literature is scant regarding CYP. Our latent class analysis broadly agrees with CLoCk 3 months postinfection findings of a (1) low and (2) multiple symptom prevalence group among PCR-test-positive CYP, where the latter was dominated by tiredness, headache, SOB and dizziness.^40^ Our qualitative work highlighted the burden of treatment and care pathways echoing findings from semistructured interviews conducted with CYP (including some from CLoCk).^35^ The long-lasting anxiety CYP with persistent PCC report echoes the global longitudinal deterioration in mental health of adolescents and young people due to the pandemic,^41^ and our findings provide context for the reported anxiety. Our qualitative work suggests that events during the pandemic and/or previous conditions might exacerbate reported symptoms, extending findings that prepandemic health is associated with PCC^42^; and underscoring the importance of considering events during and prior to the pandemic in relation to PCC symptomology. Future research linking CLoCk to prepandemic and postpandemic routinely collected data will help elucidate whether and how pre-existing vulnerabilities interact with SARS-CoV-2 symptom patterns to affect subsequent educational and health outcomes.

In conclusion, we address a methodological gap and opportunity to harness the strengths of both quantitative and qualitative analysis approaches to address ongoing challenges posed by PCC in CYP. We found that CYP with persistent PCC have poor mental health which does not improve over time. This may stem from initial education concerns and more long-term concerns around restriction relaxation. Tiredness was reported often quantitatively, but mentioned only once qualitatively, requiring further investigation. Some symptoms (eg, chills) vary over time while others, like SOB, persist for a subgroup. Findings are important for planning service provision, with both qualitative and quantitative findings warranting equal attention. Even CYP who report less frequent symptoms discuss long-lasting anxiety and symptom impact, highlighting even a few symptoms could be impairing. A nuanced understanding of symptoms experienced and their impact on physical/mental health is crucial for developing personalised intervention and management strategies to help CYP navigate their condition.

## Supplementary material

10.1136/bmjpo-2025-003634 online supplemental table 1

## Data Availability

Data are available in a public, open access repository.
