# Supplementary material for: Children and young people with persistent post-COVID-19 condition over 24 months: a mixed-methods study
Source: BMJ Paediatr Open. 2025 Oct 21;9(1):e003634. doi: 10.1136/bmjpo-2025-003634 (PMC12551525; doi:10.1136/bmjpo-2025-003634)
Supplement: online supplemental table 1 [file bmjpo-9-1-s001.docx]

| Supplementary Table 1: Demographics, symptoms, doctor consultations^¥^ and SDQ "caseness"^€^ (N (%) or Median (25th, 75th centile)), stratified by PCR-test positive CYP (i) persistently meeting the PCC research definition between 3- and 24-months post-testing (N=68) and (ii) never meeting the PCC research definition between 3- and 24-months post-testing (N=490)* | | | |
| --- | --- | --- | --- |
|  | Persistently meeting PCC definition  (N=68) | Never meeting PCC definition  (N=490) | p-value  (chi-squared test) |
| ***Demographics*** |  |  |  |
| Age at PCR testing (years) |  |  |  |
| 11-14 | 19 (27.9) | 238 (48.6) | <0.01 |
| 15-17 | 49 (72.1) | 252 (51.4) |  |
| Sex at birth |  |  |  |
| Female | 56 (82.4) | 307 (62.7) | <0.01 |
| Male | 12 (17.6) | 183 (37.4) |  |
| IMD quintile |  |  |  |
| 1 (Most deprived) | 10 (14.7) | 78 (15.9) | 0.15 |
| 2 | 16 (23.5) | 105 (21.4) |  |
| 3 | 18 (26.5) | 77 (15.7) |  |
| 4 | 12 (17.6) | 93 (19.0) |  |
| 5 (Least deprived) | 12 (17.6) | 137 (28.0) |  |
| Region of residence |  |  |  |
| Southeast | 13 (19.1) | 86 (17.6) | 0.39^β^ |
| London | 12 (17.6) | 68 (13.9) |  |
| Southwest | 12 (17.6) | 44 (9.0) |  |
| East of England | 10 (14.7) | 70 (14.3) |  |
| West Midlands | 8 (11.8) | 58 (11.8) |  |
| Northwest | 6 (8.8) | 60 (12.2) |  |
| Northeast | 1 (1.5) | 20 (4.1) |  |
| East Midlands | 4 (5.9) | 51 (10.4) |  |
| Yorkshire and The Humber | 2 (2.9) | 33 (6.7) |  |
| Ethnicity |  |  |  |
| White | 55 (80.9) | 369 (75.3) | 0.62^β^ |
| Asian/Asian British | 7 (10.3) | 74 (15.1) |  |
| Mixed/other | 5 (7.4) | 25 (5.1) |  |
| Black/African/Caribbean | 1 (1.5) | 19 (3.9) |  |
| Prefer not to say | 0 (0) | 3 (0.6) |  |
| ***Number of symptoms reported:*** |  | |  |
| 3-months post-testing | 5 (3,6) | 0 (0,2) | <0.01^α^ |
| 6-months post-testing | 5 (3,7) | 0 (0,1) | <0.01^α^ |
| 12-months post-testing | 6 (4,8.5) | 0 (0,1) | <0.01^α^ |
| 24-months post-testing | 5 (3.5,8) | 0 (0,1) | <0.01^α^ |
| ***Doctor consultation about COVID-19***^¥^ |  |  |  |
| No | 49 (72.1) | 483 (98.6) | <0.01^β^ |
| Yes | 19 (27.9) | 7 (1.4) |  |
| ***Frequency of SDQ “caseness”*** ^€^  3-to-24-months post-testing |  |  |  |
| 0 | 14 (20.59) | 400 (81.63) | <0.01 |
| 1-3 | 26 (38.24) | 82 (16.73) |  |
| 4 | 28 (41.18) | 8 (1.63) |  |
| CYP: Children and young people; IMD: Index of Multiple Deprivation; PCC: Post-COVID-19 Condition; SDQ: Strengths and Difficulties Questionnaire  ^¥^at all follow-ups CYP were asked whether they/their parents had contacted their doctor about their COVID-19  ^€^SDQ “caseness” was determined using established cut-offs for self-reported SDQ total scores[1]; those scoring ≥18 (total difficulties) were classified as a “case” (i.e., having emotional or behavioural difficulties). The number of times (0, 1-3, or 4) CYP reached “caseness” over the 24-month period post-testing was examined.  *of the 943 CYP that responded to questionnaires at 3-, 6-, 12- and 24-months post-testing: 68 meet the PCC definition at all four time-points, 490 never meet the definition and the remaining 385 meet the definition between 1 and 3 times (see[2] for more details)  ^β^p-value from Fisher’s exact test  ^α^p-value from Kruskal-Wallis rank test | | | |

**References**

1. Goodman R, SDQ Team. Scoring the Strengths and Difficulties Questionnaire (SDQ) [Internet]. 2022 [cited 2025 Aug 13]. Available from: <https://www.sdqinfo.org/py/sdqinfo/c0.py>
2. Rojas NK, Shafran R, Stephenson T, Richards-Belle A, Ortega-Martin M, Dalrymple E, Heyman I, Newlands F, McOwat K, Simmons R, CLoCk Consortium, Pinto Pereira SM. Symptom variation up to two years post-SARS-CoV-2 infection in Children and Young People: Results from the Children and young people with Long Covid (CLoCk) study [submitted]. BMC Infect Dis.
