# Supplementary material for: Children and young people with persistent post-COVID-19 condition over 24 months: a mixed-methods study
Source: BMJ Paediatr Open. 2025 Oct 21;9(1):e003634. doi: 10.1136/bmjpo-2025-003634 (PMC12551525; doi:10.1136/bmjpo-2025-003634)
Supplement: Supplementary file 2 [file bmjpo-2025-003634.reviewer_comments.pdf]

PEER REVIEW HISTORY

BMJ Paediatrics Open publishes all reviews undertaken for accepted manuscripts. Reviewers are asked to complete a checklist review form and are provided with free text boxes to elaborate on their assessment. These free text comments are reproduced below.

ARTICLE DETAILS

**TITLE (PROVISIONAL)**	Children and young people with persistent Post-COVID-19 Condition over 24 months: a mixed methods study
**AUTHORS**	Ortega-Martin, Esther; Richards-Belle, Alvin; newlands, fiona; Shafran, Roz; Stephenson, Terence; Rojas, Natalia; Batura, Neha; Buszewicz, Marta; Dalrymple, Emma; Heyman, Isobel; Consortium, CLoCk; Pinto Pereira, Snehal

VERSION 1 - REVIEW

**REVIEWER NAME**	Nicole Toepfner
**REVIEWER AFFILIATION**	Technische Universität Dresden
**REVIEWER CONFLICT OF INTEREST**	N/A
**AI DISCLOSURE**	No
**DATE REVIEW RETURNED**	03-Jun-2025

**GENERAL COMMENTS**	It is a well-ritten manucript on the persistence of self-reported PCC symptoms up to 2 years after PCR positive COVID-19.<br>A small group of children (n=68) is included without a control group. The manuscript further lacks important questions such as an evaluation if the assessed symptomes affected school participation, led to e. g. pain medication, more health care visits. ME/CFS criteria were not specifically evaluated or analysed as such. Additionally, the evaluation of characterics of the focused group is limited (older, female, white). As the results on tiredness also varied between quantitative and qualitative analysis the value of this finding remains open.<br>Therefore, overall less new evidence is provided. However, it is an important topic and consequences of the COVID-19 pandemic still affect societies. I would suggest at least adding the comparison with a control group for more information.<br>Are other items such as school participation and/or „more objective“ characteristics available? Then please add them in correlation to the self-reported symptoms of both groups.<br>How many of the 68 adolescence also have suspicion of ME/CFS or psychiatric diagnosis/treatment? Was postexertional malaise evaluated, if so – please add.<br>Please add the total number out of which the 68 adolescence where selected.<br>Please point out the resulting evidence from this study more clear.

VERSION 1 – AUTHOR RESPONSE

Reviewer: 1

It is a well-written manuscript on the persistence of self-reported PCC symptoms up to 2 years after PCR positive COVID-19.

1. A small group of children (n=68) is included without a control group.

Response: Regarding the small sample size, we acknowledge this as a study limitation on page 17, where we now say: “Our sample size was relatively small …, limiting our analysis power.”. Later (in the same paragraph) we also say: “Our qualitative analysis was limited to examining responses from CYP who filled in free-text at all time-points, and we acknowledge that this is a small, selective subgroup ….”

Regarding the lack of a control group, we are now explicit about this in the introduction (page 5) where we say: “This study was explicitly designed as an exploratory investigation to provide in-depth understanding of symptom subgroups, impairments, and lived experiences of CYP who persistently meet the research definition of PCC over a 24-month period. Importantly, a comparator group of CYP who did not persistently meet the PCC definition was not included, as the focus was on capturing and understanding experiences of those persistently affected.” We also emphasise this on page 18: “As the work presented here is exploratory, we did not compare our findings to a group of CYP who did not experience persistent PCC.” Nonetheless, we appreciate the importance of comparison and in response to point #5 below, we have added in Supplementary Table 1 comparing CYP persistently meeting the PCC definition to those never meeting the PCC definition on demographics, symptoms, doctor consultations and mental health (see response to point #5 below for more details).

2. The manuscript further lacks important questions such as an evaluation if the assessed symptoms affected school participation, led to e. g. pain medication, more health care visits.

Response: We agree that understanding the impact of persistent PCC on school participation (e.g., attendance and educational outcomes) and health-related consequences (e.g., use of pain medication, increased healthcare utilisation) is crucial. However, these specific outcome data are not available within our current dataset. For this reason, we are in the process of linking the CLoCk study data to routinely collected records from the Department of Education and Hospital Episode Statistics. We aim to address these questions in future analyses. We have now added the following to the discussion to acknowledge this important research gap (page 19): “Future research linking CLoCk to pre- and post-pandemic routinely collected data will help elucidate whether and how pre-existing vulnerabilities interact with SARS-CoV-2 symptom patterns to affect subsequent educational and health outcomes.”

3. ME/CFS criteria were not specifically evaluated or analysed as such.

Response: We agree that evaluating ME/CFS in those experiencing PCC is extremely important and is an area of ongoing research (see for example Franziska at al DOI: 10.1016/j.eclinm.2023.102146 - in adults). To date, there is a limited literature on the overlap between ME/CFS and PCC in CYP. Currently, we do not have the data to examine this ourselves. Nonetheless, it is important to consider,

and we now say (page 18) “Future studies should examine tiredness due to PCC in more detail, including its overlap with Chronic Fatigue Syndrome...”

4. Additionally, the evaluation of characterics of the focused group is limited (older, female, white). As the results on tiredness also varied between quantitative and qualitative analysis the value of this finding remains open. Therefore, overall less new evidence is provided.

Response: Regarding the three areas of concern raised:

i. Re: focused group examined – we acknowledge this as a study limitation on page 17, where we now say: “Our qualitative analysis was limited to examining responses from CYP who filled in free-text at all time-points, and we acknowledge that this is a small, selective subgroup (all female, mostly older CYP and White ethnicity).” We also now explicitly state the implications of such selection: “… generalisability of findings also warrants consideration. For example, our findings may not be applicable to … other subgroups of CYP within England (e.g., of Black/African/Caribbean ethnicity).” Of additional note, extrapolating findings from the CLoCk sample to the general population of CYP in England is an area of ongoing research. See for example BMC Med Res Methodol 24, 134 (2024) (DOI: 10.1186/s12874-024-02219-0), which has been applied in subsequent sentiment analysis of free-text responses (see Journal of Medical Internet Research, 27, Article e63634 DOI:10.2196/63634).

ii. Re: lack of triangulation of findings concerning tiredness - we have edited the text on page 16 to provide reasons for this: “Several factors may explain this discrepancy. It is possible that although CYP experienced tiredness, it was not perceived as debilitating or attributed to COVID infection/associated lockdowns. Alternatively, tiredness may have been so pervasive that it was normalised and thus not considered noteworthy. Finally, as participants were not systematically probed about specific symptoms, experiences of tiredness may not have been fully captured qualitatively.” We have also edited the “considerations” column of Table 3 to provide these three points that might explain the discrepancy between qualitative and quantitative findings regarding tiredness.

iii. Re: lack of new evidence – we have edited the discussion to be more explicit on the added value and novelty of our study. For example, on page 18:

a. “However, the added value of this current manuscript is that we describe, in depth, symptom clusters, impairments, health and experiences of CYP persistently meeting the PCC definition over a 24-month period. Crucially, we also describe how these symptom clusters, impairments and experiences evolved over time.”

b. “Here we extend this observation to demonstrate that there are two different PCC symptom profile subgroups both reporting high levels of tiredness.”

c. We also point out the added value with relevant references: “Previous studies have identified PCC symptom clusters in adults[38,39] but the literature is scant regarding CYP. Our latent class analysis…”

Finally, we have added to the final paragraph on page 19: “In conclusion, we address a methodological gap and opportunity to harness the strengths of both quantitative and qualitative analysis approaches to address ongoing challenges posed by PCC in CYP.”

5. However, it is an important topic and consequences of the COVID-19 pandemic still affect societies. I would suggest at least adding the comparison with a control group for more information.

Response: At the reviewer’s request, we have provided a new Table (Supplementary Table 1), which we explain on page 7: “To determine the representativeness of our analytical sample we examined whether CYP (following a positive PCR-test) who persistently met the PCC definition over 24-months differed from those who never met the PCC definition over the same timeframe. We used chi-squared, Fisher’s exact or Kruskal-Wallis rank tests as appropriate.” We then describe the comparison between these two groups on page 9: “Compared to CYP who provided data at all four time-points but never met the PCC definition, the analytical sample was older and more likely to be female (Supplementary Table 1). The two groups were otherwise similar in terms of deprivation (IMD quintile), region of residence, and ethnicity.”

6. Are other items such as school participation and/or “more objective” characteristics available? Then please add them in correlation to the self-reported symptoms of both groups.

Response: See response to point #2 above regarding information on school participation and corresponding edits made to the manuscript. While no objective measures are currently available, we do have self-reported data on (i) whether CYP (or their parents) ever contacted a doctor about their COVID-19 and (ii) validated scales. One such scale is the Strengths and Difficulties Questionnaire (SDQ), which is used to measure emotional and behavioural difficulties. We have now added this information into the manuscript. For example, see page 6 for an explanation of the SDQ: “Validated scales, including the EuroQol Five Dimensions Youth (EQ-5D-Y) questionnaire[26] (a measure of health-related quality of life) and the Strengths and Difficulties Questionnaire[27] (SDQ; used to measure emotional and behavioural difficulties), were also collected.”. We also provide data on the SDQ and contact with a doctor about COVID-19 in Supplementary Table 1. See also response to point #7 below.

7. How many of the 68 adolescence also have suspicion of ME/CFS or psychiatric diagnosis/treatment?

Response: As explained in response #3 we do not have the data to examine ME/CFS. Relatedly, we do not have data on psychiatric diagnosis/treatment. However, we do have data on (i) whether CYP (or their parents) ever contacted a doctor about their COVID-19 and (ii) the validated Strengths and Difficulties Questionnaire (SDQ). Using this data, we now show in Supplementary Table 1 that 19/68 (27.9%) of CYP with chronic PCC contacted a doctor about their COVID-19 and 28/68 (41%) were ‘chronic SDQ cases’.

8. Was postexertional malaise evaluated, if so – please add.

Response: This was not evaluated, but is an important step for future research, which we now say on page 18: “Future studies should examine tiredness due to PCC in more detail, including its overlap with Chronic Fatigue Syndrome, and potentially assess post-exertional malaise.”.

9. Please add the total number out of which the 68 adolescence where selected.

Response: We now explain on page 5: “This study focused on CYP who PCR-tested positive for SARS-CoV-2 in January, February or March 2021; 23,048 test-positive CYP were invited to participate and 3,321 enrolled. Of these, 943 CYP provided data at 3-, 6-, 12- and 24-months post-testing with 68 persistently meeting the PCC definition (see below) at all four time-points.” We also

provide more details in a footnote to (new) supplementary Table 1: “of the 943 CYP that responded to questionnaires at 3-, 6-, 12- and 24-months post-testing: 68 meet the PCC definition at all four time-points, 490 never meet the definition and the remaining 385 meet the definition between 1 and 3 times.”

10. Please point out the resulting evidence from this study more clear.

Response: we have edited the discussion to be more explicit on the resulting evidence from our study. For example, on page 18:

- “However, the added value of this current manuscript is that we describe, in depth, symptom clusters, impairments, health and experiences of CYP persistently meeting the PCC definition over a 24-month period. Crucially, we also describe how these symptom clusters, impairments and experiences evolved over time.”

- “Here we extend this observation to demonstrate that there are two different PCC symptom profile subgroups both reporting high levels of tiredness.”

- We also point out the added value with relevant references: “Previous studies have identified PCC symptom clusters in adults[38,39] but the literature is scant regarding CYP. Our latent class analysis…”

- Finally, we have added to the final paragraph on page 19: “In conclusion, we address a methodological gap and opportunity to harness the strengths of both quantitative and qualitative analysis approaches to address ongoing challenges posed by PCC in CYP.”
